# The prognostic value of *IL10* and *TNF alpha* functional polymorphisms in premenopausal early-stage breast cancer patients

**DOI:** 10.1186/s12863-015-0234-8

**Published:** 2015-06-26

**Authors:** Erika Korobeinikova, Dana Myrzaliyeva, Rasa Ugenskiene, Danguole Raulinaityte, Jurgita Gedminaite, Kastytis Smigelskas, Elona Juozaityte

**Affiliations:** Oncology Institute, Lithuanian University of Health Sciences, Eiveniu str. 2, LT-50009 Kaunas, Lithuania; Oncology Research Laboratory, Oncology Institute, Lithuanian University of Health Sciences, Eiveniu str. 2, LT-50009 Kaunas, Lithuania; Health Research Institute, Lithuanian University of Health Sciences, Betonuotoju str. 4-9, LT-52371 Kaunas, Lithuania

**Keywords:** Breast cancer, Prognosis, *IL10*, *TNFalpha*, Single nucleotide polymorphism, SNP

## Abstract

**Background:**

Interleukin-10 and tumor necrosis factor α play an important role in breast carcinogenesis. Genes, encoding those two cytokines, contain single nucleotide polymorphisms, which are associated with differential levels of gene transcription. This study analyzes single nucleotide polymorphisms in *interleukin 10* and *tumor necrosis factor α* genes and their contribution to breast cancer phenotype, lymph node status and survival in a group of young Lithuanian women with early-stage breast cancer patients.

**Results:**

We genotyped 100 premenopausal Eastern European (Lithuanian) patients with stage I-II breast cancer, ≤50 years old at the time of diagnosis, for *interleukin 10* -592A > C, −819C > T and -1082A > G and *tumor necrosis factor α* -308G > A single nucleotide polymorphisms in the gene promoter region. We used the polymerase chain reaction, namely a restriction fragment length polymorphism method, for a SNP analysis. All genotypes were in Hardy-Weinberg equilibrium and had the same distribution as the HapMap CEU population. Holders of *IL10* -592A > C heterozygous *IL10* -592 AC genotype had a higher probability of estrogen receptor positive breast cancer phenotype than homozygous variants (P = 0.017). Phased ACC haplotype of *IL10* polymorphisms was associated with younger age of diagnosis (P = 0.017). Of all the tested single nucleotide polymorphisms, only *TNFα* -308G > A has revealed a prognostic capability for breast cancer survival. GA genotype carriers, compared to GG, showed a significant disadvantage in progression-free survival (P = 0.005, adjusted hazard ratio (HR) = 4.631, 95 % confidence interval (CI) = 1.587 – 13.512), metastasis-free survival (P = 0.010, HR = 4.708, 95 % CI = 1.445 – 15.345) and overall survival (P = 0.037, HR = 4.829, 95 % CI = 1.098 – 21.243).

**Conclusions:**

According to our data, *IL10* -1082A > G, −819 T > C, −592A > C polymorphisms and phased haplotypes have not revealed a prognostic value for breast cancer. On the contrary, the *TNF*α -308 polymorphism might modulate the risk and contribute to the identification of patients at a higher risk of breast cancer recurrence, metastasis and worse overall survival among young Lithuanian early-stage breast cancer patients.

## Background

Breast cancer (BC) comprises about one fourth of all female cancers worldwide. Despite new diagnostic and treatment options, roughly 30 % of early-stage patients will progress to metastatic disease [1]. Experimental genetic research and genome-wide association studies have significantly improved our understanding of complex BC biology, the process of the disease development in particular. However, it is equally important to extend our knowledge on the course the disease takes by following its development to identify patients who are likely to have a more aggressive disease and to tailor their treatment.

It has been well established that several cytokines, including Interleukin-10 (IL-10) and Tumor Necrosis Factor α (TNFα), have a crucial role in a coordinated manner in breast carcinogenesis [2]. Genes, encoding IL-10 and TNFα cytokines, contain several nucleotide variations, namely single nucleotide polymorphisms (SNPs), which are associated with different levels of gene transcription and determine interindividual differences in IL-10 and TNFα production [3, 4].

Over the recent years, three functional SNPs, constituting substitutions of a single bases upstream of the transcriptional start site of *IL10* gene, have been investigated: *IL10* adenine (A) to guanin (G) substitution at -1082 bp (rs1800896), *IL10* thymin (T) to cytosine (C) substitution at -819 bp (rs1800871) and *IL10* A to C substitution at -592 bp (rs1800872) [5]. These SNPs affect transcriptional activity, leading to alterations in gene expression that influence IL-10 production [3, 4]. They are strongly linked together and present three major haplotypes, ATA, ACC, and GCC, which are associated with low, medium and high levels of *IL10* expression respectively. GCC individuals secrete on average two or three times more IL-10 than wild type ATA individuals [6]. It was proven by several authors that IL-10 levels in blood samples of breast cancer patients correlate directly with the clinical stage of the disease [7, 8].

SNP in the promoter region of the *TNFα* locus has been identified at position −308, which also showed that it involves the replacement of G by A [9]. *TNFα* -308G > A GA and AA genotypes lead to a higher rate of *TNFα* gene transcription than wild type GG genotype *in vitro* [10]. High plasma TNFα levels in cancer patients are associated with a poor disease outcome [11]. *TNFα* expression significantly increases at the advanced stages of breast cancer [12]. The TNFα protein induces the expression of adhesion molecules, facilitating the invasion of metastatic tumor cells [13]. Several studies have shown a close link between *TNFα* -308G > A polymorphism and breast cancer risk [14].

Some investigators found genetic evidence for association between *IL10* -1082A > G, −819 T > C, −592A > C and *TNFα* -308G > A polymorphisms and breast cancer progression in different ethnic populations [8, 15]. However, the data is not consistent [5], poorly differentiated in terms of ethnicity, cancer stage, age etc. This study, therefore, aimed to investigate the relationship between functional SNPs in *IL10* and *TNFα* and BC clinicopathologic features and survival in a highly homogeneous group of patients, taking into account age, race and stage of the disease at the time of diagnosis to identify whether these genetic determinants may be important for BC prognosis.

## Materials and Methods

### Patients

Adult female primary stage I-II BC patients (≤50 years old at the time of diagnosis) in premenopausal state (n = 100) were involved in this research. Women with other malignant tumors, poor performance status, other significant comorbidities and/or incomplete medical documentation were not included in the study. Adjuvant therapy was chosen by clinicians, based on pathomorphological characteristics and validated prognosis factors, according to national recommendations. All the study subjects were Eastern European (Lithuanian).

### Specimen Characteristics and Assay Methods

Samples were collected in 2009–2014. Genomic DNA was extracted from peripheral blood leukocytes by using the commercially available DNA extraction kit (Thermo Fisher Scientific), with regard to the manufacturer’s protocol. A *IL10* gene promoter polymorphisms analysis was performed by using a polymerase chain reaction-based restriction fragment length polymorphism method (PCR-RFLP).

*IL10* gene regions including -592A > C, −819C > T and -1082A > G polymorphic sites were amplified by using primers reported by Liu *et al*. [16]. For -592C > A and -819C > T polymorphisms, the same reaction mixture composition was employed. Briefly, PCR reaction was carried out in a total volume of 25 μl, containing 1x DreamTaq standard buffer, template DNA, 0.24 μM of each primer, 200 μM of each dNTP and 1.25 U of DreamTaq DNA polymerase (Thermo Fisher Scientific, Waltham, MA, USA) with annealing at 63 °C and 58 °C for -592C > A and -819C > T polymorphisms respectively. PCR reaction conditions for *Il10* gene -1082G > A polymorphism were slightly modified by adding 4.0 mM MgCl_2_, 4 % DMSO and changing the annealing temperature to 56 °C.

Following PCR, the amplicons underwent digestion with different restriction endonucleases. *Rsa*I restriction endonuclease (Thermo Fisher Scientific Baltics, Lithuania) was used for a -592C > A polymorphism analysis. In the presence of A allele, *Rsa*I yielded 175 and 237 bp fragments, while C allele remained uncut (412 bp). *Mae*III restriction endonuclease was implemented for a -819C > T polymorphism detection. The presence of *Mae*III restriction site indicated C allele (125 and 84 bp fragments), while T allele remained undigested (209 bp). For a -1082G > A polymorphism identification, PCR products were incubated with *Mnl*I enzyme (Thermo Fisher Scientific Baltics, Lithuania), which cut G allele into 106 and 33 bp fragments, while A allele remained uncut (139 bp). The results were visualized on 2 % agarose gel containing ethidium bromide.

The primer sequences for a *TNFα* -308G > A fragment amplification were reported by Kaur *et al.* [17]. PCR reaction was carried out in a total volume of 25 μl, containing 1x DreamTaq standard buffer, template DNA, 0.24 μM of each primer, 200 μM of each dNTP, 4.0 mM MgCl_2_, 4 % DMSO and 1.25 U of DreamTaq DNA polymerase (Thermo Fisher Scientific, Waltham, MA, USA). The annealing temperature for *TNFα* -308G > A polymorphism was 63 °C.

Restriction endonuclease *Nco*I was used to detect the *TNFα* -308G > A polymorphism. With regard to *TNFα* -308G > A promoter polymorphism, G allele was represented by 87 bp and 20 bp fragments, while A allele by 107 bp fragment. Restriction endonuclease products were separated on 3 % agarose gels containing ethiduim bromide.

### Study Design

A prospective cohort study was conducted at the Oncology Institute of Lithuanian University of Health Sciences. A full ethical approval was obtained from the Kaunas Regional Bioethics Committee (protocol number BE-2-13) and the Lithuanian Data Protection Agency (protocol number 2R-2246). Every subject has signed informed consent forms before commencing the study. For a case selection, the information of the period of 2001–2011 about primarily BC patients was retrieved from the Pathology Department at the Hospital of Lithuanian University of Health Sciences. The patients were matched by disease stage, age of disease onset and menopausal status. The patients' clinicopathological information was obtained from their medical files. The patients were monitored according to the clinical monitoring protocol till 1^st^ May 2014. The median follow-up was 70 months. Disease progression was defined as a local breast cancer recurrence in the affected breast and distant metastases in visceral organs, skeleton, skin or the central nervous system. Date of cancer histological verification was considered as time zero for survival analysis. The SNPs selected for associations with the known breast cancer prognostic factors and cancer progression were as follows: *IL10* -1082A > G, −819 T > C, −592A > C, and *TNFα* -308G > A. This study was conducted adhering to recommendations for tumor marker prognostic studies [18, 19].

### Statistical Analysis

A Hardy–Weinberg Equilibrium for the genotype distribution of the selected SNPs was tested in all cases by using the Pearson X^2^ test and the Fisher Exact test. To evaluate if the frequencies of alleles and genotypes correspond with the data of earlier studies, we retrieved information from a population of the International HapMap project of Northern Europeans from Utah (CEU) (HapMap Data rel 28 Phasell + III, August10, on NCBI B36 assembly, dbSNP b126, http://hapmap.ncbi.nlm.nih.gov). *IL10* haplotypes were inferred from promoter *IL10* SNPs by Bayesian methods as implemented in the Phase software (version 2.1; Department of Statistics, University of Washington, Seattle, Washington, USA) [20, 21]. For demonstration of linkage disquelibrium (LD) SNP block was performed using Haploview v4.1. The block followed the haplotype block definition of solid spine of LD as implemented in Haploview v4.1 [22]. Statistical analyses were performed by using SPSS® for Windows software version 20.0 (Released 2011. Armonk, NY: IBM Corp.). P value of less than 0.05 was considered significant. Bonferroni-corrected alpha level was used in association analysis for multiple comparisons. The Pearson Chi-square or the Fisher Exact test was used for categorical data. Associations between genotype and disease-free survival (DFS), metastasis-free survival (MFS) and overall survival (OS) were investigated by using Kaplan-Meier’s method and estimated by performing a log-rank test. The association analysis included genotype, allelic models and haplotype model for *IL10* SNPs. Cox regression models were used to adjust the analysis for potential confounders. SNPs were re-evaluated in a model adjusted for the known breast cancer prognostic values, which included age group (30–40 years, 41–50 years), tumor size (T1, 2), lymph node status (N0, 1), histological grade (G1, 2, 3) and intrinsic subtype (Luminal A, Luminal B, HER2 enriched, Basal-like), by carrying out a multivariate regression analysis as well as computing odds ratios and 95 % confidence intervals (95 % CI).

## Results

### Sample Characteristics

**Fig. 1:**
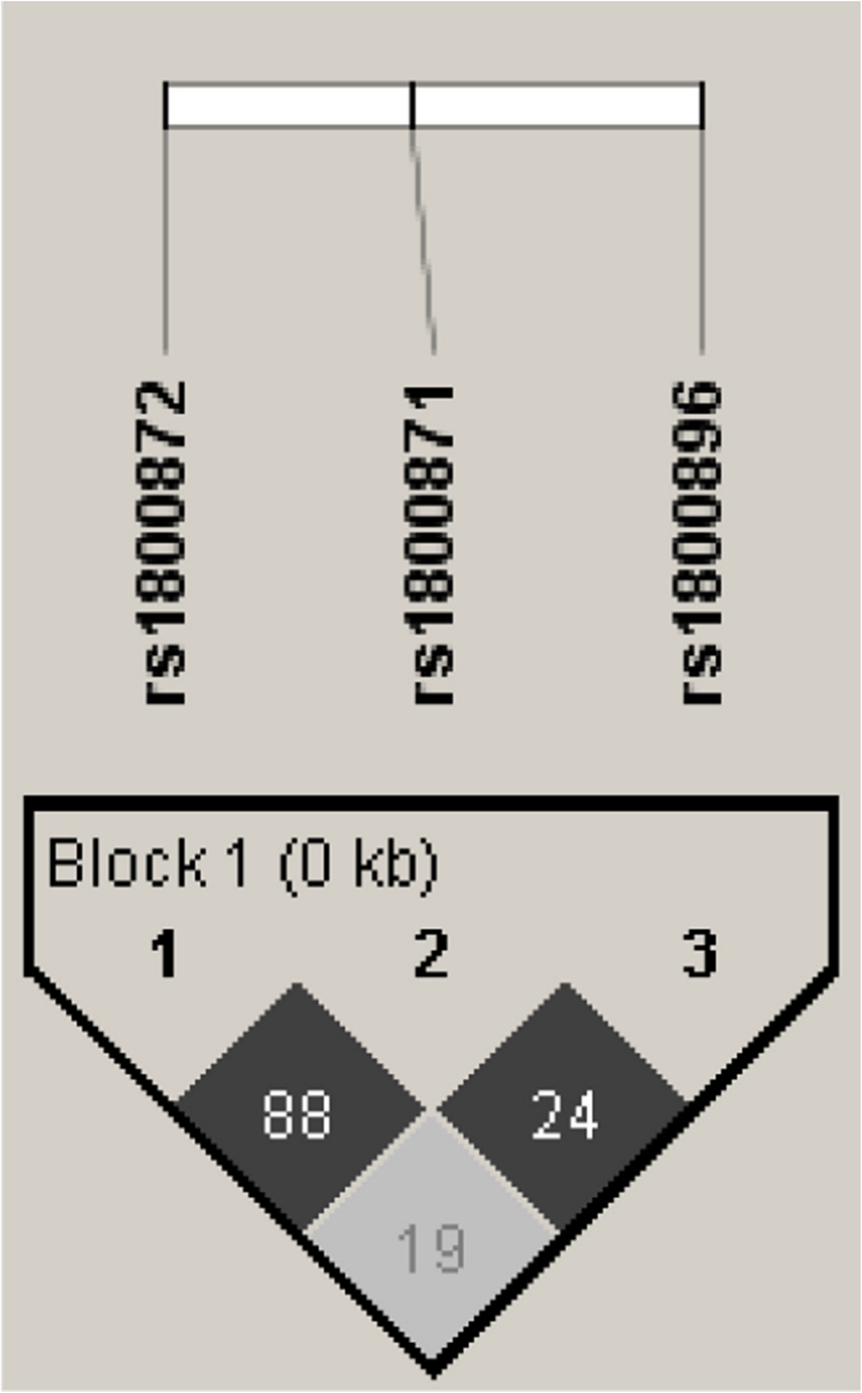
Linkage disequilibrium and haplotype block. Numerical values are given of r2 values, whereas the colors are given to encode D’ (dark grey encodes strong evidence of LD). Block followed the haplotype block definition of solid spine of LD as implemented in the Haploview v.4.1 [22]

The analysis included 100 primary, young, premenopausal, early stage breast cancer patients. The frequency data for clinical and tumor biological factors is shown in Table 1. All the patients were genotyped for a panel of four SNPs: *IL10* -1082A > G, −819 T > C, −592A > C, and *TNFα* -308G > A. The genotypes were found to be in Hardy-Weinberg equilibrium in all the four SNPs. A strong LD was confirmed for *IL10* -819 T allele with IL10 -592A allele and IL10 -819 C allele with IL10 -592C allele (Fig. 1). Our cohort statistically has the same genotype distribution as the HapMap CEU population. The allele and genotype frequencies determined in our study and, for comparison, HapMap CEU population are shown in Table 2.

**Table 1:** Frequencies of clinical and tumor biological factors

Age group	
30-40years	34/100
41-50years	66/100
Tumor size (pathologic)	
T1	64/100
T2	36/100
Lymph node involvement (pathologic)	
N0	55/100
N1	45/100
Grade	
G1	9/100
G2	62/100
G3	29/100
Estrogen receptors (ER)	
ER positive	57/100
ER negative	43/100
Progestin receptors (PR)	
PR positive	48/100
PR negative	52/100
Human epidermal growth factor receptor 2 (HER2)	
HER2 positive	28/100
HER2 negative	72/100
Intrinsic subtype	
Luminal A	46/100
Luminal B	18/100
HER2 enriched	10/100
‘Basal-like’	26/100

**Table 2:** Allele and genotype frequencies of the *IL10* and *TNFα* gene promoter regions. Data from our study and HapMap CEU population

Gene	Polymorphism	Allele and genotype frequencies				
		(HAPMAP CEU allele and genotype frequencies data)				
*TNFα*	−308 G > A (rs1800629)	G	A	GG	GA	AA
		0.900	0.100	0.83	0.14	0.03
		(0.827)	(0.173)	(0.877)	(0.123)	(0)
*IL10*	−1082 A > G (rs1800896)	A	G	AA	GA	GG
		0.415	0.585	0.20	0.43	0.37
		(0.469)	(0.531)	(0.212)	(0.513)	(0.274)
	−819 T > C (rs1800871)	T	C	CC	CT	TT
		0.255	0.745	0.58	0.33	0.09
		(0.179)	(0.821)	(0.661)	(0.321)	(0.018)
	−592 A > C (rs1800872)	C	A	CC	AC	AA
		0.720	0.280	0.54	0.36	0.10
		(0.788)	(0.212)	(0.628)	(0.319)	(0.053)

### Inferential Analysis

The estimation of associations between the known BC prognostic variables and the studied polymorphisms in genotype model revealed a significant link between *IL10* -592A > C SNP and ER status (P = 0.017). The carriers of heterozygous AC genotype had 3.231 times higher probability of ER positive BC phenotype than CC genotype carriers (95 % CI 1.282 - 8.141; P = 0.011) and 4.500 times higher than AA genotype carriers (95 % CI 1.032 - 19.630; P = 0.037). The allelic model showed no close relationships of *IL10* -592A > C SNP with tumor biological and clinical prognostic factors. The analysis of *IL10* -1082A > G, *IL10* -819 T > C and *TNFα* -308G > A SNPs in both genotype and allelic models showed no significant links with clinicopathological features.

Phasing revealed three main, well-known haplotypes, namely GCC, ACC and ATA. A few uncommon haplotypes were confirmed (ACA and GCA), which were not included in the association analysis. The haplotype frequency data are shown in Table 3. The haplotype analysis confirmed the ACC haplotype connection with younger age (30–40 years) of disease onset (P = 0.017). Non-carriers of ACC haplotype 2.951 times more frequently belonged to older patient subgroup (41 – 50 years) than carriers (95 % CI 1.198 – 7.273; P = 0.017). GCC and ATA haplotypes did not show any significant associations with the known breast cancer prognostic factors.

**Table 3:** Relative haplotype frequencies of *IL10* promoter polymorphism on the total number of chromosomes

Haplotype	Frequencies (valid percent*)
GCC	41 %
ACC	32.8 %
ATA	26.2 %

*2 rare ACA and 3 GCA haplotypes were not included in the haplotype association analysis

### Survival Analysis

**Fig. 2:**
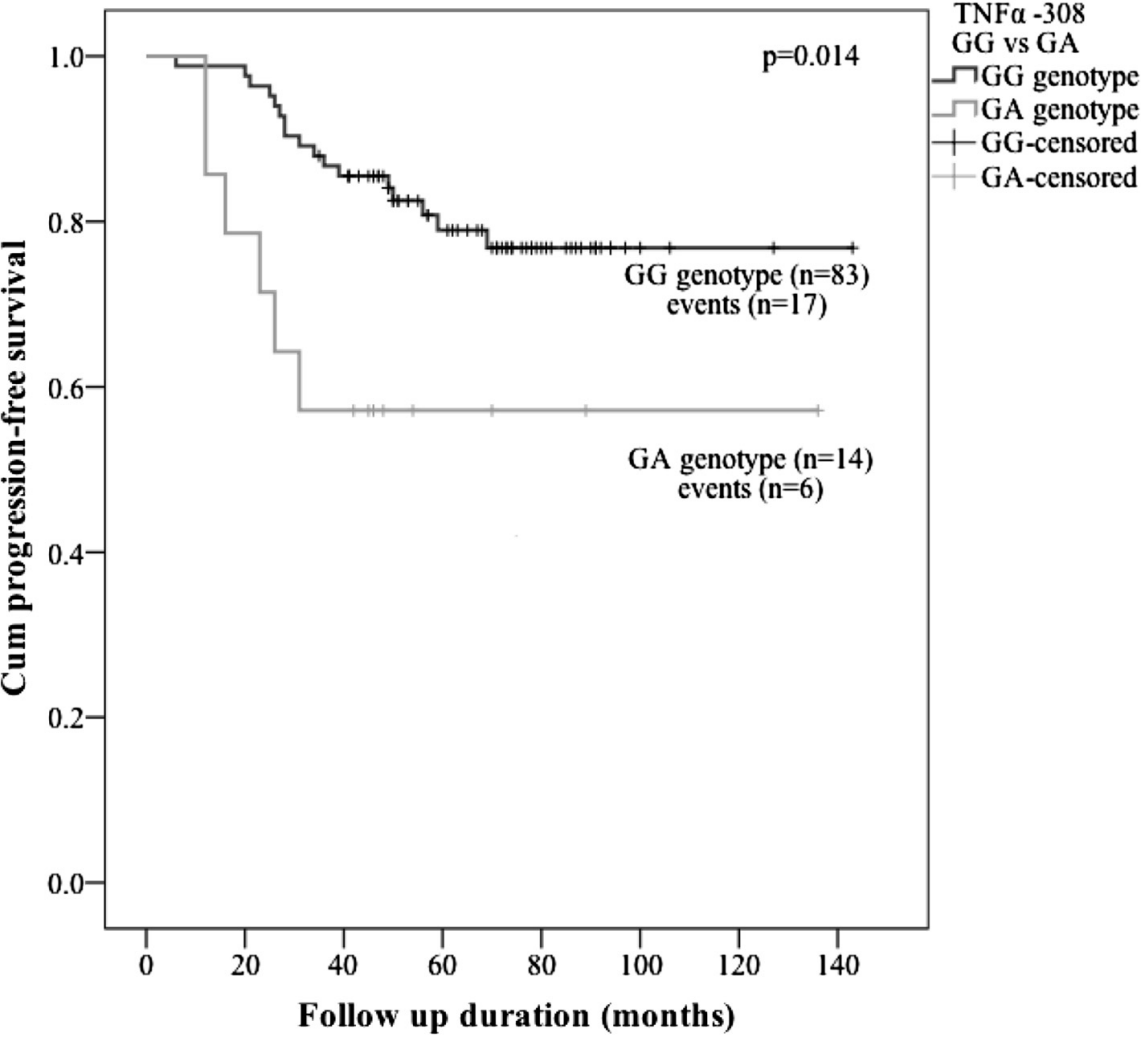
Kaplan–Meier curves for progression-free survival of *TNFα* -308G > A polymorphism GG and GA genotypes

In the median follow-up time of 70 months (range 28–157), progression was observed for 24 patients. 76 cases were censored. Of those who progressed, 20 had distant metastases. 14 patients with progressive disease died, all due to cancer related death. The data of Cox’s proportional hazards regression analysis is shown in Table 4. Kaplan-Meier and Cox's regression analysis did not reveal any significant relationships between the analyzed *IL10* -1082A > G, −819 T > C, −592A > C SNPs and phased haplotypes and PFS, MFS and OS in our study. Cox’s regression analysis of *TNFα* -308G > A SPN has shown a significant disadvantage of GA genotype *vs.* two others in PFS (P = 0.020, hazard ratio (HR) = 3.049, 95 % CI = 1.195-7.778) and MFS (P = 0.045, HR = 2.819, 95 % CI = 1.021-7.780). During a further analysis of this SNP, we evaluated only the major GG genotype *vs.* heterozygous GA because of a small number of AA genotypes in our population. GG genotype of the *TNFα* -308G > A polymorphism was significantly associated with a longer PFS by carrying out the Kaplan-Meier analysis, which is graphically shown in Fig. 2 (P = 0.014). Mean PFS was 119 months in GG genotype group (95 % CI 108–129) *vs.* 86 months in GA genotype group (95 % CI 56–116).

**Table 4:** Cox’s univariate model. Unajusted hazard ratios for PFS, MFS, OS with each of the SNPs in genotype, allelic and haplotype model

Reference SNP ID		Genotype/allele /haplotype	*n*	Progression-free survival		Metastasis-free survival		Overall survival	
				Multivariate	*P* value	Multivariate	*P* value	Multivariate	*P* value
				Hazard ratio (CI)		Hazard ratio (CI)		Hazard ratio (CI)	
*IL10* -1082A > G	Genotype model	GG	37	1	0.317	1	0.456	1	0.288
		GA	43	3.168	0.131	2.580	0.221	1.524	0.606
				(0.709-14.157)		(0.565-11.779)		(0.307-7.565)	
		AA	20	2.840	0.182	2.493	0.248	3.138	0.168
				(0.613-13.169)		(0.592-11.753)		(0.617-15.951)	
	Allelic model	A allele non carriers	63	1		1		1	
		A allele carriers	37	3.020	0.135	0.819	0.663	0.431	0.128
				(0.708-12.885)		(0.334-2.008)		(0.145-1.276)	
		G allele non carriers	80	1		1		1	
		G allele carriers	20	0.852	0.708	2.541	0.211	2.021	0.359
				(0.367-1.974)		(0.589-10.953)		(0.450-9.086)	
*IL10* -819 T > C	Genotype model	CC	58	1	0.695	1	0.905	1	0.357
		CT	33	1.456	0.396	1.234	0.665	2.029	0.242
				(0.612-3.466)		(0.477-3.188)		(0.620-6.643)	
		TT	9	1.109	0.892	1.176	0.833	2.516	0.253
				(0.248-4.963)		(0.260-5.314)		(0.518-12.221)	
	Allelic model	C allele non carriers	91	1		1		1	
		C allele carriers	9	1.042	0.956	0.918	0.909	0.502	0.370
				(0.244-4.447)		(0.213-3.960)		(0.111-2.265)	
		T allele non carriers	42	1		1		1	
		T allele carriers	58	1.378	0.444	1.220	0.658	2.157	0.161
				(0.606-3.131)		(0.505-2.950)		(0.736-6.322)	
*IL10* -592A > C	Genotype model	CC	54	1	0.877	1	0.995	1	0.427
		AC	36	1.131	0.637	1.048	0.923	1.849	0.311
				(0.517-2.935)		(0.405-2.709)		(0.563-6.073)	
		AA	10	0.941	0.941	1.011	0.989	2.411	0.276
				(0.211-4.231)		(0.224-4.570)		(0.495-11.728)	
	Allelic model	C allele non carriers	90	1		1		1	
		C allele carriers	10	1.152	0.848	1.007	0.992	0.512	0.384
				(0.270-4.920)		(0.233-4.347)		(0.114-2.311)	
		A allele non carriers	46	1		1		1	
		A allele carriers	54	1.168	0.712	1.039	0.932	1.992	0.211
				(0.513-2.656)		(0.430-2.515)		(0.676-5.863)	
*TNFα* -308G > A	Genotype model	GG	83	1	0.066	1	0.135	1	0.163
		GA	14	3.049*	0.020	2.819*	0.045	3.096	0.057
				(1.195-7.778)		(1.021-7.780)		(0.967-9.909)	
		AA	3	N.c.	0.981	N.c.	0.982	N.c.	0.989
	Allelic model	G allele non carriers	97	1		1		1	
		G allele carriers	3	21.241	0.548	21.252	0.992	21.069	0.725
				(0.001; >1000)		(0.001; >1000)		(0.001; >1000)	
		A allele non carriers	17	1		1		1	
		A allele carriers	83	2.256	0.088	2.904	0.153	2.643	0.102
				(0.887-5.738)		(0.760-5.768)		(0.825-8.471)	
*IL10*	Haplotype model	GCC non carriers	43	1		1		1	
		GCC carriers	57	1.502	0.353	1.483	0.401	1.143	0.805
				(0.637-3.544)		(0.592-3.718)		(0.396-3.300)	
		ACC non carriers	38	1		1		1	
		ACC carriers	62	0.890	0,785	0.854	0.730	0.456	0.154
				(0.384-2.063)		(0.348-2.095)		(0.155-1.343)	
		ATA non carriers	58	1		1		1	
		ATA carriers	42	1.374	0.448	1.214	0.667	2.104	0.174
				(0.605-3.121)		(0.502-2.935)		(0.720-6.150)	

*Significant associations.

N.c. – no cases

**Fig. 3:**
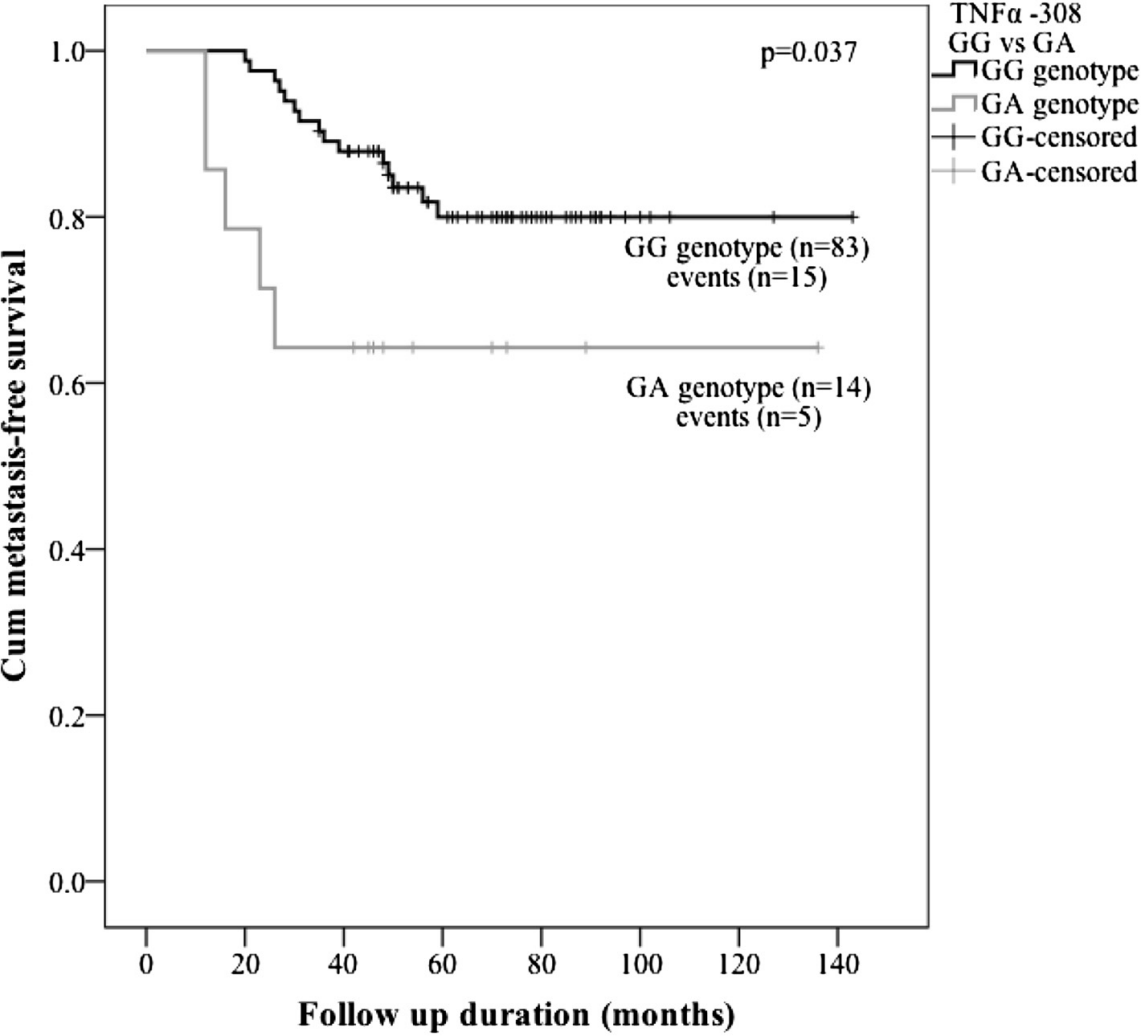
Kaplan–Meier curves for metastasis-free survival of *TNFα* -308G > A polymorphism GG and GA genotypes

**Fig. 4:**
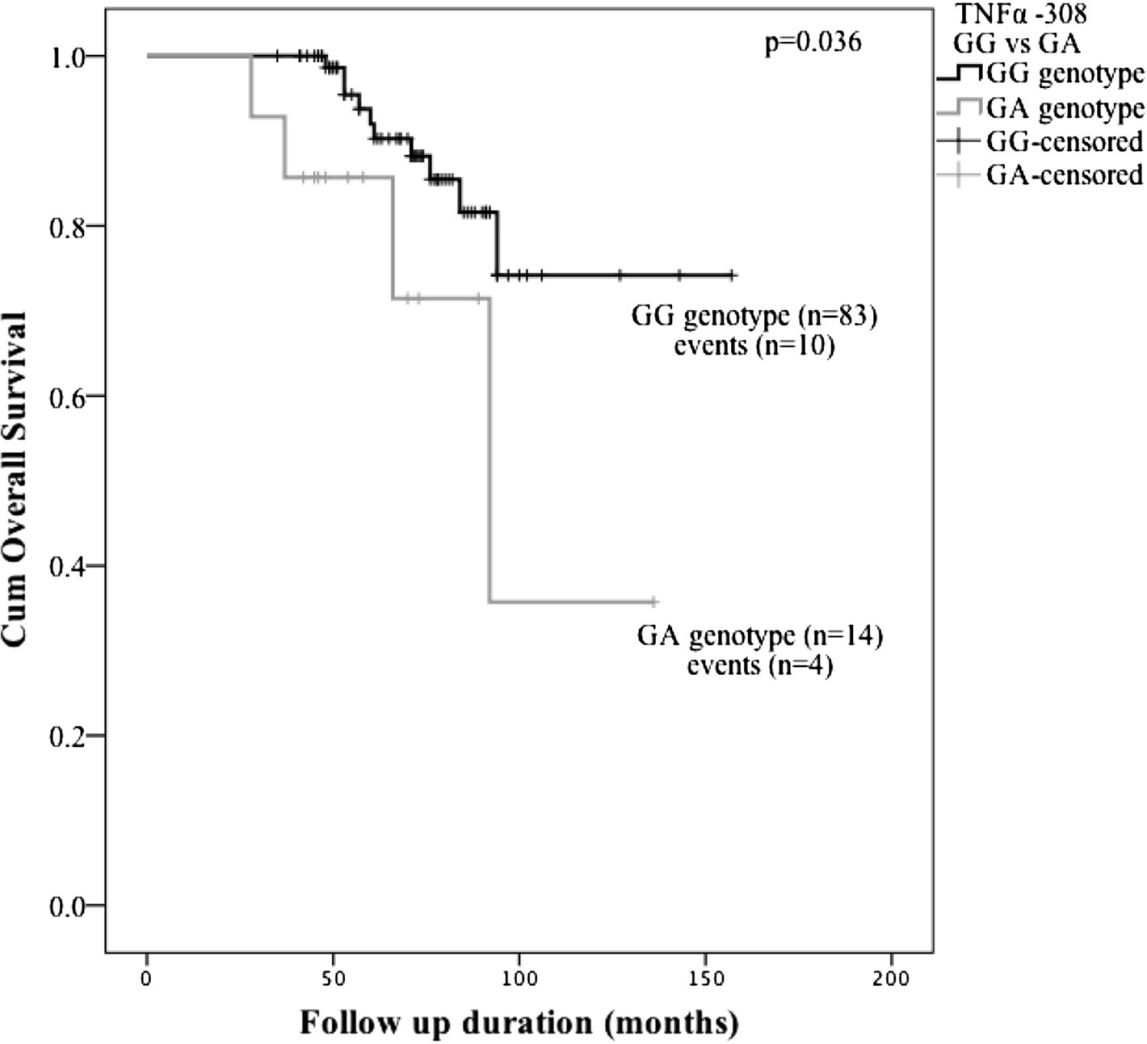
Kaplan–Meier curves for overall survival of *TNFα* -308G > A polymorphism GG and GA genotypes

As far as MFS is concerned, the benefit of GG genotype *vs.* GA was also demonstrated by Kaplan-Meier curves (P = 0.037, Fig. 3). The mean time of MFS was 122 months in GG genotype group (95 % CI 112–132) *vs*. 93,7 months in GA genotype group (95 % CI 64–124). The period of follow-up is rather short to evaluate OS differences, however, preliminary data also shows unequal survival between GG and GA genotypes of *TNFα* -308G > A SNP (P = 0.036) (Fig. 4).

After adjusting to age group, tumor size, histological grade, lymph node status, ER, PR, HER2 status and intrinsic subtype, *TNFα* GA genotype of *TNFα* -308G > A SNP remained a significant negative prognostic factor for PFS (P = 0.005, HR = 4.631, 95 % CI = 1.587-13.512), MFS (P = 0.010, HR = 4.708, 95 % CI =1.445 – 15.345) and OS (P = 0.037, HR = 4.829, 95 % CI =1.098 – 21.243), which is shown in Table 5.

**Table 5:** Cox’s multivariable model. Adjusted hazard ratios for PFS, MFS, OS with each of the known BC prognostic factor and *TNFα* -308G > A

Variable		Progression-free survival		Metastasis-free survival		Overall survival	
		Hazard ratio (95 % CI)	P value	Hazard ratio (95 % CI)	P value	Hazard ratio (95 % CI)	P value
*TNFα* -308G > A	GG genotype	1		1		1	
	GA genotype	4.631*	0.005	4.708*	0.010	4.829*	0.037
		(1.587-13.512)		(1.445-15.345)		(1.098-21.243)	
Age group	41-50 years	1		1		1	
	30-40 years	1.451	0.403	1.407	0.481	1.014	0.983
		(0.606-3.477)		(0.544-3.639)		(0.283-3.634)	
Tumor size (pathologic)	T1	1		1		1	
	T2	1.039	0.934	0.749	0.555	0.577	0.425
		(0.419-2.581)		(0.286-1.960)		(0.149-2.233)	
Lymph node involvement (pathologic)	N0	1		1		1	
	N1	1.876	0.192	2.349	0.199	1.346	0.628
		(0.729-4.828)		(0.829-6.659)		(0.405-4.480)	
Grade	G1	1	0.962	1	0.751	1	0.629
	G2	1.268	0.825	1.080	0.944	0.542	0.598
		(0.154-10.449)		(0.127-9.184)		(0.056-5.268)	
	G3	1.378	0.783	0.972	0.981	0.293	0.375
		(0.141-13.477)		(0.095-9.965)		(0.019-4.412)	
Intrinsic subtype	Luminal B	1	0.191	1	0.140	1	0.119
	Luminal A	4.095	0.178	3.329	0.225	1.380	0.780
		(0.526-31.892)		(0.419-26.433)		(0.144-13.257)	
	‘Basal-like’	3.872	0.233	3.248	0.317	3.966	0.285
		(0.420-35.739)		(0.324-32.593)		(0.318-49.534)	
	HER2 overexpression	9.874*	0.044	10.177*	0.043	6.426	0.112
		(1.068-91.312)		(1.080-95.880)		(0.646-63.903)	

*Significant associations.

## Discussion

In this prospective cohort study of 100 premenopausal female patients with early-stage breast cancer, we investigated associations between functional SNPs in *IL10* and *TNFα* genes, previously implicated in breast cancer occurrence, spread and survival. We found that the SNP genotype frequency data of *IL10* -1082A > G, −819 T > C, −592A > C and *TNFα* -308G > A correspond to HAPMAP project CEU population data and obey the Hardy-Weinberg law of genetic equilibrium.

*IL10* -1082A > G polymorphism did not show any significant correlation with tumor characteristics, lymph node status and the course of the disease. In the Asian population, Kong *et al.* showed a larger tumor size for those with AA genotype at position −1082 in comparison to other genotypes and a significantly lower lymph node involvement in patients harboring at least one G allele of this SNP [15]. However, supporting our results, none of the reported European studies showed this SNP to be associated with tumor phenotype or survival [8, 23–26]. Despite the fact that in earlier studies the −1082 G allele (which had also been related to higher *IL10* expression [10]) was associated with a lower breast cancer risk [27], it seems not to have a major impact on a further course of the disease in our study.

Carriers of *IL10* -592A > C heterozygote AC genotype and *IL10* -819 T > C CT genotype had a higher probability of ER positive BC type than homozygote variants. Our data conflict with other authors who did not find any associations of these SNPs with ER status [15, 23, 28]. Furthermore, in the Chinese population, Jingyan *et al.* [29] did not reveal any significant locus–locus interaction between ER coding genes and *IL10* -1082, *IL10* -819, or *IL10* -592 SNPs, which could explain associations of these SNPs with ER status. However, there is lack of data on this topic in the European population in literature.

Our results of the *IL10* -819 T > C and -592A > C SNP association analysis with other known BC prognostic factors and survival confirm a few other authors’ findings, i. e. those SNPs are neither related with clinicopathological tumor data (except ER status as mentioned earlier) nor with PFS, MFS or OS [15, 23, 25, 30]. However, our data contradict the study of Slattery *et al.* [31], who have recently showed the *IL10* -819 TT genotype as a potential factor for lower cancer risk with OR of 0.79 and Gerger *et al.* [8], who revealed A-allele of the *IL10* -592C > A polymorphism to have a prognostic value of the reduced DFS with 1.45 risk ratio; yet, controversially, this allele was earlier proved to be linked with a lower BC risk [28].

Due to strong linkage disequilibrium between *IL10* -819 T > C and -592C > A SNPs, the presence of ATA haplotype could be determined by analyzing the -592C > A polymorphism: the -592A allele indicated the presence of the ATA haplotype, whereas the -592C allele indicated its absence. Phasing revealed three main, well-known haplotypes, namely GCC (41 %), ACC (32.8 %) and ATA (26.2 %). An association between ACC haplotype and younger age of disease onset was found. In the Asian population, as earlier reported [15], the authors discovered ATA haplotype to be associated with a significantly increased risk of lymph node metastasis and a higher tumor size at the time of diagnosis. We did not reproduce these results in the Lithuanian population. ATA haplotype in our study did not show any distinction from other haplotypes in the association and survival analysis. The literature on survival differences among breast cancer patients with different *IL10* haplotypes is extremely poor. Data from one small Iranian study support our results [32].

Functional *IL10* polymorphisms are of particular interest when describing BC because IL-10 has both potentially cancer-promoting immunosuppressive and potentially cancer-inhibiting antiangiogenic properties. Despite the fact that Langsenlehner *et al.* [28] revealed that genetically programmed low *IL10* expression may be protective in susceptibility to breast cancer, according to our data it seems to have no importance to a further development of the disease.

*TNFα* -308G > A SNP has showed the greatest prognostic potential for BC of all the analyzed SNPs. GA genotype (earlier reported as a high plasma TNF producer) in BC patients was found to be significantly associated with a poor disease outcome, while wild GG genotype, usually linked to low plasma TNF levels, was associated with a better prognosis. The multivariate regression model indicated *TNFα* -308G > A SNP as an independent prognostic factor for PFS, MFS and OS. As a biological background for these results may serve the fact, that TNFα protein induces an epithelial-mesenchymal transition, namely the process through which cancer cells at the invasive front of primary tumors undergo a phenotypic conversion to invade and metastasize through the circulation and generate a metastatic lesion at distant tissues or organs [33]. A chronic and consistent presence of TNF*α* in tumors leads to procancerous consequences in many malignant diseases [34]. *TNFα* is overexpressed in approximately 90 % of patients with recurrent disease [12]. Similarly, Mestiri *et al*. discovered that the low producer *TNF*α -308G > A AA genotype was often associated with the reduced DFS and/or overall survival in patients with breast cancer [35]. Azmy *et al.* revealed that the carriage of low producer -308A allele might predispose to a more aggressive disease [36]. A study in Tunisia concluded that individuals with the AA genotype were more susceptible to and had worse prognoses in BC [32]. An Italian study did not demonstrate any association between *TNFα* -308G > A polymorphism genotypes and BC [27]. Murray *et al. *[25] failed to confirm TNF alpha polymorphisms as a potential indicator for time to recurrence in Caucasians, African Americans and Hispanics. Controversially, a meta-analysis of Caucasian and Asian ethnicities reported by Fang *et al.* [14] suggested that the G allele of *TNFα* -308G > A is a risk factor for breast cancer development, especially for Caucasians. A contrasting nature of the results of all these studies may be accounted for by sampling error or by differences in ethnicity of patient groups.

We take into consideration a limited sample size, the risk of other confounders and nonrandom sampling. However, this study supports the relevance of *TNFα* germline polymorphisms to BC prognosis and our findings hold promise for further investigations, preferable on larger cohorts from different ethnic origins.

## Conclusions

In conclusion, our findings suggest that *IL10* -1082A > G, −819 T > C, −592A > C SNPs have no sufficient data of association with the prognosis of BC. Contrary, the *TNF*α -308 polymorphism might modulate the risk and could contribute to the identification of patients at a higher risk of BC recurrence, metastasis and overall survival in Lithuanian early-stage breast cancer patients. To confirm the validity and utility of these polymorphisms as clinical prognostic biomarkers, future studies of a wider European population are needed.

## Abbreviations

BC: Breast cancer
*IL10*: *Interleukin 10* gene
IL-10: Interleukin 10 protein
*TNFα*: *Tumor necrosis factor alpha* gene
TNF*α*: Tumor necrosis factor protein
SNP: Single nucleotide polymorphism
A: Adenine
G: Guanine
T: Thymine
C: Cytosine
PCR: Polymerase chain reaction
CEU: Northern Europeans from Utah
DFS: Disease-free survival
MFS: Metastasis-free survival
OS: Overall survival
CI: Confidence interval
ER: Estrogen receptor
PR: Progesterone receptor
LD: Linkage disequilibrium
